# Fusion *in situ* versus reduction for spondylolisthesis treatment: grading the evidence through a meta-analysis

**DOI:** 10.1042/BSR20192888

**Published:** 2020-06-24

**Authors:** Rui He, Guo-lin Tang, Kun Chen, Zheng-liang Luo, Xifu Shang

**Affiliations:** Department of Orthopedics, The First Affiliated Hospital of USTC, Division of Life Sciences and Medicine, University of Science and Technology of China, Hefei 230001, Anhui, P.R. China

**Keywords:** fusion in situ, meta analysis, reduction, spondylolisthesis

## Abstract

**Purpose:** During surgical procedure on lumbar spondylolisthesis, the role of reducing slip remains controversial. The purpose of the present study was to compare fusion *in situ* with reduction in clinical and radiographic outcomes.

**Methods:** A literature research was performed at PubMed, Embase, Web of Science, and Cochrane Library. After screening by two authors, ten articles were brought into this meta-analysis finally, and the quality was evaluated by the modified Newcastle–Ottawa Scale (NOS). Isthmic, moderate, and serious spondylolisthesis were all analyzed separately. Sensitivity analyses were performed for high-quality studies, and the publication bias was evaluated by the funnel plot.

**Results:** Most criteria did not have statistical differences between reduction and fusion *in situ* groups. However, in reduction group, the union rate was significantly higher (*P*=0.008), the slippage was much improved (*P*<0.001) and the hospital stay was much shorter comparing to no-reduction group (*P*<0.001). Subgroup analysis (containing moderate and serious slip, or isthmic spondylolisthesis) and sensitivity analysis were all consistent with original ones, and the funnel plot indicated no obvious publication bias in this meta-analysis.

**Conclusions:** Both reduction and fusion *in situ* for lumbar spondylolisthesis were related with good clinical results. Reduction led to higher rate of fusion, better radiographic slippage, and shorter hospital stay. After sufficient decompression, reduction did not incur additional risk of neurologic impairment compared with fusion *in situ*.

## Introduction

Spondylolisthesis is defined as the forward slippage of one vertebra on another. Stability reconstruction and neural decompression have been widely considered as the effective treatment for spondylolisthesis [1]. To achieve these aims, various fusion methods with different surgical approaches have been used, such as anterior lumbar interbody fusion (ALIF), posterior lumbar interbody fusion (PLIF), and transforaminal lumbar interbody fusion (TLIF) [2]. In recent decade, minimally invasive TLIF (MIS-TLIF) has been developed, this operation could result in less blood loss, less soft-tissue injury, and earlier rehabilitation [3].

**Figure 1:**
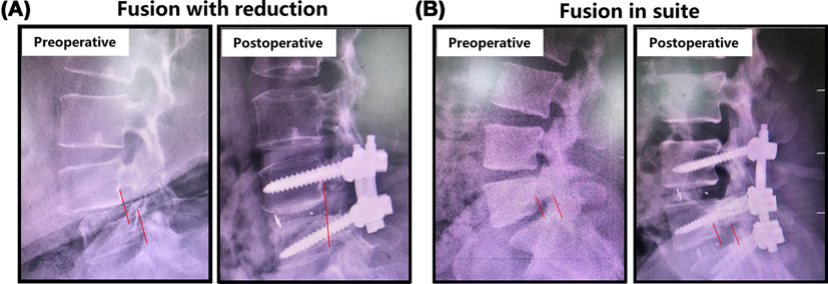
Schematic diagram of fusion *in situ* and fusion with reduction (**A**) Fusion *in situ*. (**B**) Fusion with reduction.

The main surgical strategies for treating spondylolisthesis include spinal fusion *in situ* and spinal fusion with reduction. Fusion *in situ* means that the vertebras will be fused ‘where it is’ with little or no correction of the spine; while fusion with reduction suggests that the slippage will be reduced or realigned during the surgery (Figure 1). Despite the evolution of surgical treatment procedures, it still remains controversial whether the management of reducing the spondylolisthesis is necessary [1,4,5].

During surgical procedure, fusion *in situ* is commonly performed with stability reconstruction and effective neural decompression. Nevertheless, compared with reduction in the slipped lumbar vertebra, it leads to higher pseudarthrosis rates and progressive parameters of the deformity, especially in patients with high-grade spondylolisthesis [6].

Reduction in the spinal anatomy and disk space height to restore the sagittal spinal balance is extended with the development of surgical techniques and instrumentation [1]. However, the reduction procedure is associated with increased risks of neurological complications, loss of reduction, and prolonged treatment time [1].

Previous systematic review has compared arthrodesis and neurologic deficits *in situ* fusion group with reduction group for high-grade spondylolisthesis [6]. However, more and more studies are published in recent years, including surgical management with MIS-TLIF [2,3,7]. More clinical and radiographic outcomes could be reviewed. Therefore, in the present study, we conducted a meta-analysis to compare different results between *in situ* fusion group and reduction group for low- and high-grade spondylolisthesis.

## Materials and methods

### Search strategy

A literature search using PubMed, Embase, Web of Science, and Cochrane Library was performed in May 2016 without restriction on time, nation, and publication types. The search strategy was: (fusion or Arthrodesis) AND (*in situ*) AND (Reduction) AND (Spondylolisthesis). In addition, the references of every selected articles were checked manually to find out if they were also related.

### Inclusion and exclusion criteria

Studies satisfying the following criteria were included for review: published studies comparing the outcomes between *in situ* arthrodesis and reduction in spondylolisthesis; randomized controlled trial (RCT) or retrospective comparative studies (cohort or case–control studies) on humans; studies that were required to provide available data to calculate the odds ratio (OR) and the corresponding 95% confidence interval (95% CI).

Correspondingly, studies were excluded if they met the following criteria: reviews, case reports, conference abstracts, and editorials; data that overlapped with previous publications. If potentially eligible studies reported overlapped data, the most comprehensive one was included in our systematic review.

### Methodological quality assessment

The methodological quality of eligible studies was evaluated following a modified 9-star system of the Newcastle–Ottawa Scale (NOS) [9]. The ‘star system’ was applied to judge each study on three broad perspectives: the selection of the study cases (four items), the comparability of the study populations (two items), and the ascertainment of either the exposure or outcome of interest (three items). Studies scoring≥7 stars were deemed as high quality. Disagreements between investigators were settled by discussion until consensus was reached.

### Data extraction

In compliance with the predefined criteria, following information was meticulously extracted independently by two reviewers from all qualified articles: surname of the primary author; year of publication; lever of evidence; patients’ number; age; Meyerding grade; comparison category and operative method. The comparable parameters were divided into primary and secondary outcomes. Primary outcomes contained clinical and radiological results; VAS score; ODI score; JOA score; patient satisfaction; union rate and complication. Patient satisfaction surveys recorded five-point Patient Subjective Outcome scores (excellent, good, fair, unchanged, worse) [8]. Radiological results included slippage, lumbar lordosis, and lumbosacral angle. Secondary outcomes contained hospital stay, blood loss, and operative time.

### Statistical analysis

The data were pooled by the Cochrane Collaboration’s Review Manager 5.3. Continuous results were presented as mean difference (MD) and with 95% CI while dichotomous outcomes presented as OR with 95% CI [10,11]. Heterogeneity among studies was evaluated by the Cochran’s Q test and the *I^2^* statistic [12]. *P*<0.1 or *I^2^* > 50% was considered to be heterogeneous. The random-effects model was used if there is heterogeneity (*P*<0.1 or *I^2^* > 50%) between studies, otherwise, the fixed-effects model was used.

Sensitivity analyses were performed for high-quality studies and funnel plots were used to screen for potential publication bias. Moreover, the moderate spondylolisthesis (Meyerding grades I, II) and severe slip (Meyerding grades III, IV) were also analyzed in subgroup, and two surgical methods were also compared in the isthmic spondylolisthesis as subgroup analysis.

## Results

### Search results

**Figure 2:**
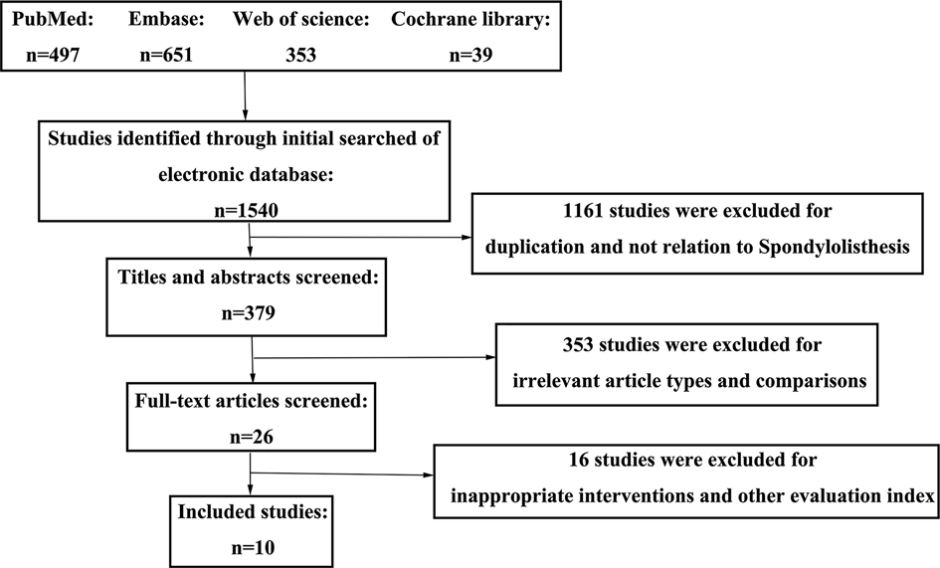
A flow diagram shows the selection process of studies

A literature research found 1540 potentially relevant articles from four databases (Figure 2). A total of 1161 studies were excluded for duplication or not related to spondylolisthesis. After screening the titles and abstracts, 353 articles were excluded for irrelevant article types or no comparison. Other 16 articles were excluded for inappropriate interventions. Finally, ten articles, including two RCTs [4,5] and eight comparative studies [1–3,7,13–16], were brought in the meta-analysis.

**Figure 3:**
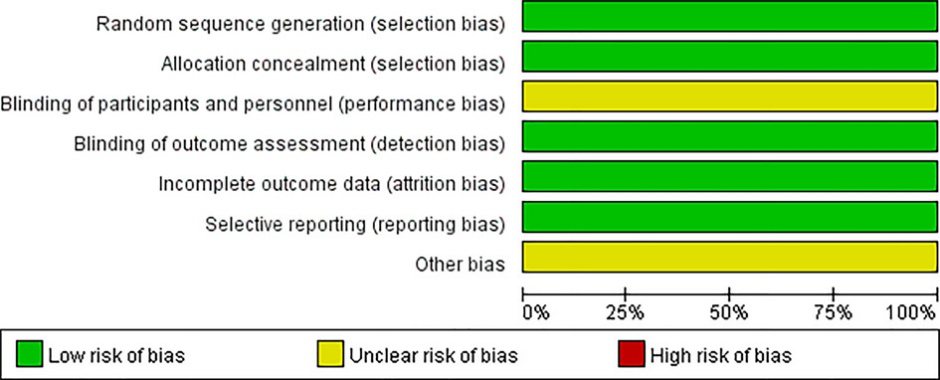
The quality of RCTs which was evaluated by the Cochrane Collaboration’s Handbook

The basic characteristics of these selected articles are shown in Table 1, and the quality of included studies, which were evaluated by modified NOS, are detailed in Table 2. The quality of RCTs, which was evaluated by the Cochrane Collaboration’s Handbook is shown in Figure 3 [17].

**Table 1:** Characteristics of included studies are showed below

Study	Level of evidence	Patient number		Age (years)	Meyerding grade	Matching*	Operative methods	Quality score
		Reduction	*In situ*					
Fan et al. [2016]	2b	24	21	50.5/50.1	I, II	1, 2, 3, 4, 5, 6	MIS-TLIF*	★★★★★★★★★
Tay et al. [2016]	2b	30	26	56.4/58.3	I, II	1, 2, 3, 5	MIS-TLIF	★★★★★★★
Scheer et al. [2015]	4	162	120	61.7/61.9	I, II, III	1	MIS-TLIF	★★★★★★
Lian et al. [2014]	1b	45	43	Mean 45.2	I, II, III	1, 2, 3, 4, 5, 6	PLIF*	RCT*
Lian et al. [2013]	1b	36	37	74.3/73.8	I, II	1, 2, 3, 4, 5, 6	PLIF	RCT
Martiniani et al. [2012]	4	10	6	Mean 19.6	III, IV	NA	PLIF	★★★★★
Gong et al. [2010]	2b	21	13	45.3/47.1	III, IV	1, 2, 5, 6	TLIF*	★★★★★★★
Poussa et al. [2006]	4	11	11	14.9/14.5	III, IV	1	PLIF/ALIF*	★★★★★★
Molinari et al. [1999]	2b	26	11	13.8/14.4	III, IV	1, 4, 5	PLIF	★★★★★★★
Muschik et al. [1997]	2b	30	29	14.0/14.0	II, III, IV	1, 2, 3, 5	ALIF	★★★★★★★

**Table 2:** Qualities of included articles were evaluated by modified NOS

Study	Selection				Comparability		Outcomes		Quality score
	Case definition	Representativeness	Selection of controls	Definition of controls	Comparable for 1, 2, 3*	Comparable for 4, 5, 6*	Assessment of outcome	Integrity of follow-up	
Fan et al. [2016]	Yes	No	Yes	Yes	Yes	Yes	Yes	Yes	★★★★★★★★
Tay et al. [2016]	Yes	No	Yes	Yes	1, 2, 3	5	Yes	Yes	★★★★★★★
Scheer et al. [2015]	Yes	No	Yes	Yes	1	No	Yes	Yes	★★★★★★
Lian et al. [2014]									RCT
Lian et al. [2013]									RCT
Martiniani et al. [2012]	Yes	No	Yes	Yes	No	No	Yes	Yes	★★★★★
Gong et al. [2010]	Yes	No	Yes	Yes	1, 2	5, 6	Yes	Yes	★★★★★★★
Poussa et al. [2006]	Yes	No	Yes	Yes	1	No	Yes	Yes	★★★★★★
Molinari et al. [1999]	Yes	No	Yes	Yes	1	4, 5	Yes	Yes	★★★★★★★
Muschik et al. [1997]	Yes	No	Yes	Yes	1, 2	5, 6	Yes	Yes	★★★★★★★

* *P*<0.05

## Meta-analysis results

### Primary outcomes

Primary outcomes were divided into clinical and radiological ones (Table 3). VAS scores, ODI scores, and JOA scores had no significant differences between reduction and fusion *in situ* groups (*P*=0.46, *P*=0.56, *P*=0.22). Expect for the JOA scores (*I^2^* = 83, *P*<0.1), there were hardly any heterogeneity in other two items (*I^2^* = 0, *P*=0.72, *I^2^* = 0, *P*=0.78). No statistical differences in patient satisfaction (*P*=0.73) and complication rates (0.97) were found. And the neuropathic symptom was also similar in both groups. Whereas the union rates were obviously higher in reduction groups (*P*=0.008). While the *I^2^* statistic and the Cochran’s Q test of satisfaction, complication and union rates were generally lower (*I^2^* = 0, *P*=0.85, *I^2^* = 25, *P*=0.22, *I^2^* = 0, *P*=0.73). Radiological outcomes contained slippage, lumbar lordosis, and lumbosacral angle. The radiographic slippage in the reduction group was significantly improved comparing to fusion *in situ* group (*P*<0.001). There were no significant differences between two groups in lumbar lordosis and lumbosacral angle (*P*=0.79, *P*=0.57). Except for the lumbosacral angle (*I^2^* = 0, *P*=0.75), the heterogeneity of the rest two items was considerably high (*I^2^* = 85, *P*<0.1, *I^2^* = 85, *P*<0.1).

**Table 3:** The results of comparison of reduction and fusion *in situ* are shown below

Outcomes of interest	Study number	Reduction patient number	*In situ* patient number	WMD/OR* (95% CI)	*P* value	Study heterogeneity			
						x^2^	df	*I^2^*,%	*P* value*
Primary outcomes									
Clinical outcomes									
VAS score	5	152	125	0.08 (−0.13, 0.29)	0.46	2.08	4	0	0.72
ODI score	4	126	114	0.66 (−1.58, 2.90)	0.56	1.08	3	0	0.78
JOA score	3	105	101	_1.28 (−3.30, 0.75)	0.22	11.58	2	83	<0.01
Satisfaction	5	150	132	1.12 (0.59, 2.13)	0.73	1.37	4	0	0.85
Union rate	5	273	217	2.06 (1.21, 3.51)	<0.05	0.63	2	0	0.73
Complication	9	385	311	0.99 (0.67, 1.47)	0.97	10.62	8	25	0.22
Neuropathic symptom	6	162	141	1.11 (0.44, 2.78)	0.83	4.95	5	0	0.42
Radiological outcomes									
Slippage [%]	7	177	160	_16.96 (−21.85, −12.06)	<0.05	39.84	6	85	<0.01
Lumbar lordosis [°]	4	122	117	_0.78 (−6.49, 4.94)	0.79	20.51	3	85	<0.01
Lumbosacral angle [°]	4	72	59	_0.73 (−3.24, 1.78)	0.57	1.21	3	0	0.75
Secondary outcomes									
Hospital stay [days]	4	237	180	_0.13 (−0.18, −0.08)	<0.05	1.18	3	0	0.76
Blood loss [ml]	7	328	266	35.01 (−4.99, 75.02)	0.09	26.18	6	77	<0.01
Operation time [min]	7	328	266	9.07 (−1.18, 19.32)	0.08	16.98	6	65	<0.01

Abbreviations: df, degree of freedom; WMD/OR, weighted MD/OR. *P<0.05

### Secondary outcomes

Four articles including 417 patients showed that the hospital stay time was shorter in reduction group compared with fusion *in situ* group, and the heterogeneity was also low (*P*<0.001, *I^2^* = 1.18, *P*=0.76) in hospital stay. There were no significant differences between two groups when compared the blood loss (*P*=0.09) and operative time (*P*=0.08). Whereas the *I^2^* statistic and the Cochran Q test were both high in blood loss (*I^2^* = 77, *P*<0.1) and operative time (*I^2^* = 65, *P*<0.1).

### Isthmic spondylolisthesis

**Figure 4:**
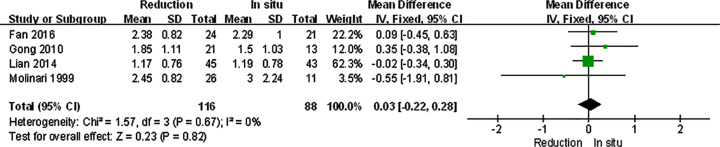
Subgroup analysis of VAS score in isthmic spondylolisthesis

**Figure 5:**
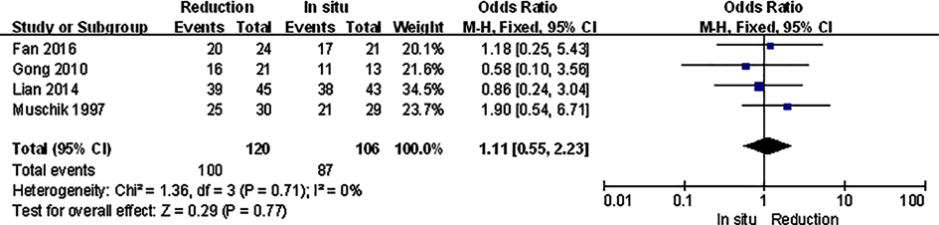
Subgroup analysis of satisfaction in isthmic spondylolisthesis

**Figure 6:**
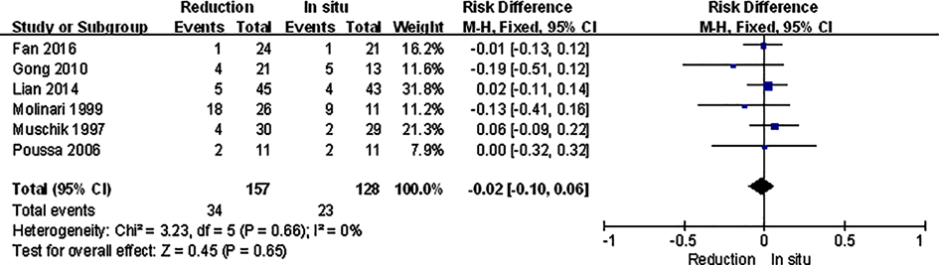
Subgroup analysis of complication in isthmic spondylolisthesis

**Figure 7:**
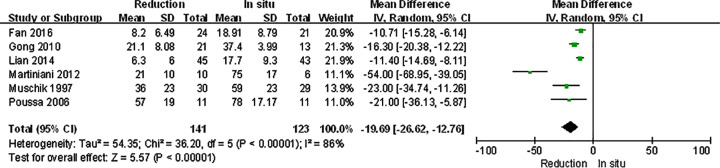
Subgroup analysis of slippage in isthmic spondylolisthesis

For isthmic spondylolisthesis patients, all the primary outcomes we analyzed were consistent with the original ones. There were no significant differences in VAS score, satisfaction, and complication, and the slippage was much better in reduction group (Figures 4–7).

### Subgroup analysis

**Figure 8:**
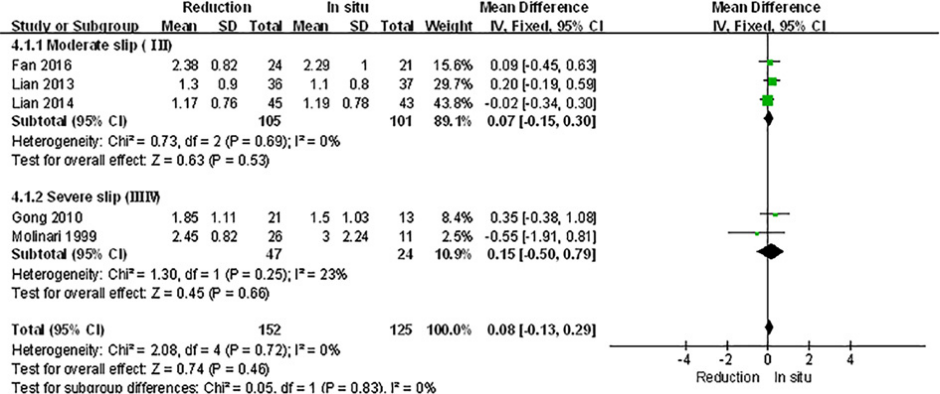
Subgroup analysis of VAS scores after spondylolisthesis were divided into moderate and severe ones

**Figure 9:**
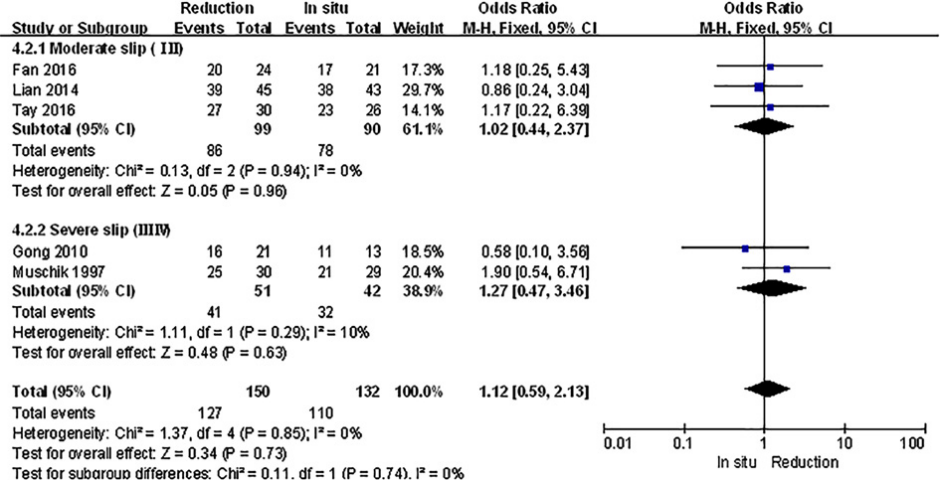
Subgroup analysis of satisfaction after spondylolisthesis were divided into moderate and severe ones

**Figure 10:**
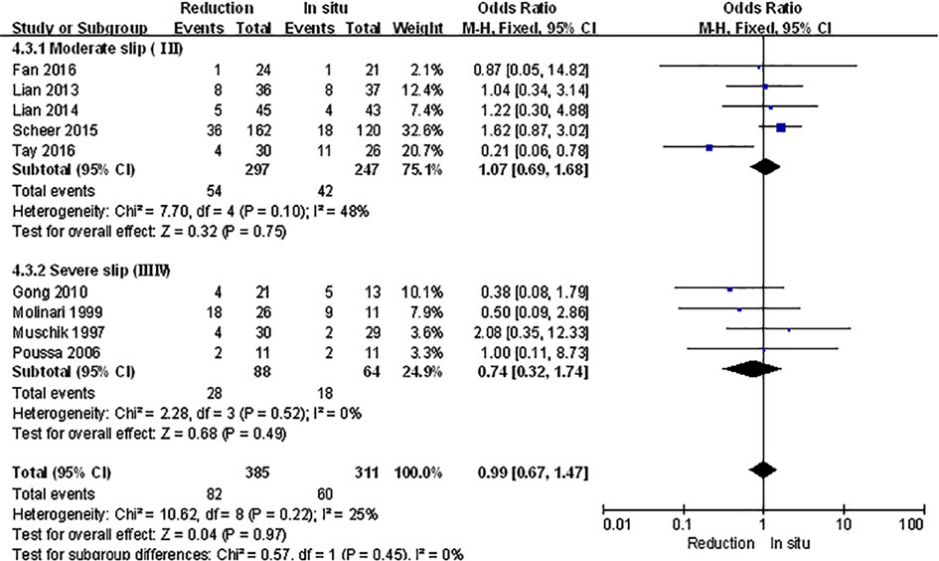
Subgroup analysis of complication after spondylolisthesis were divided into moderate and severe ones

**Figure 11:**
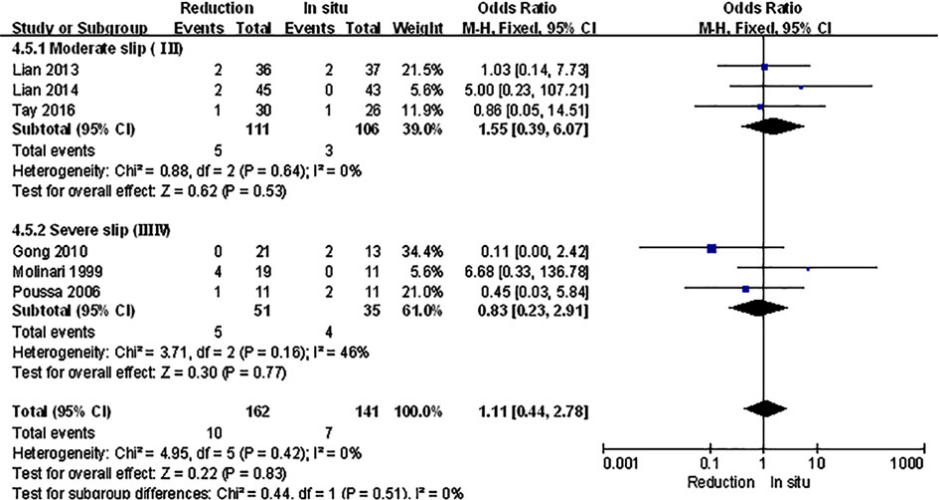
Subgroup analysis of neuropathic symptom after spondylolisthesis were divided into moderate and severe ones

**Figure 12:**
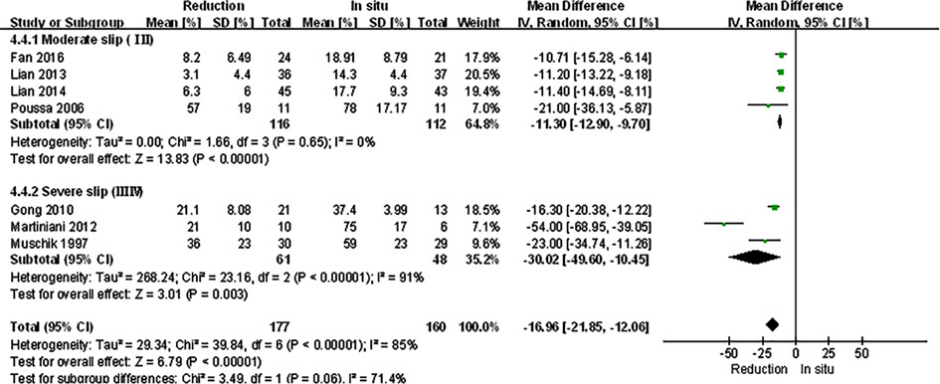
Subgroup analysis of slippage after spondylolisthesis were divided into moderate and severe ones

The spondylolisthesis patients were divided into different subgroups according to Meyerding grade: moderate (Meyerding grades I, II) and serious slip (Meyerding grades III, IV). The analysis was performed in order to compare the primary outcomes between reduction and fusion *in situ* groups in each subgroups. All the items in subgroups, including VAS scores (Figure 8), satisfaction (Figure 9), complication (Figure 10), neuropathic symptom (Figure 11), and slippage (Figure 12), were similar to the original outcomes. In radiological outcomes, the slippage was much better in reduction group. And there were no statistical differences in all other three indexes.

### Sensitivity analysis and publication bias

Two RCTs and five high-quality retrospective studies were brought into sensitivity analysis (Table 4). Different from the previous results, the lumbar lordosis was greater in reduction group (*P*<0.001) and the length of hospital stay was similar in two groups (*P*=0.2). All other items were nearly the same as the original ones.

**Table 4:** Sensitivity analysis comparison of reduction and fusion *in situ* are shown below

Outcomes of interest	Study number	Reduction patient number	*In situ* patient number	WMD/OR* (95% CI)	*P* value	Study heterogeneity			
						x^2^	df	*I^2^*,%	*P* value*
Primary outcomes									
Clinical outcomes									
VAS score	5	152	125	0.08 (−0.13, 0.29)	0.46	2.08	4	0	0.72
ODI score	4	126	114	0.66 (−1.58, 2.90)	0.56	1.08	3	0	0.78
JOA score	3	105	101	−1.28 (−3.30, 0.75)	0.22	11.58	2	83	<0.01
Satisfaction	5	150	132	1.12 (0.59, 2.13)	0.73	1.37	4	0	0.85
Union rate	5	273	217	2.06 (1.21, 3.51)	0.008	0.63	2	0	0.73
Complication	7	212	180	0.67 (0.39, 1.16)	0.15	6.54	6	8	0.37
Neuropathic symptom	5	151	130	1.28 (0.47, 3.50)	0.63	4.46	4	10	0.35
Radiological outcomes									
Slippage [%]	5	156	143	−12.70 (−15.28, −10.12)	<0.001	8.67	4	54	0.07
Lumbar lordosis [°]	3	111	106	4.00 (2.20, 5.81)	<0.001	3.98	2	50	0.14
Lumbosacral angle [°]	2	51	42	−0.36 (−2.99, 2.27)	0.79	0.06	1	0	0.81
Secondary outcomes									
Hospital stay [days]	3	75	60	−0.77 (−1.92, 0.39)	0.2	0.03	2	0	0.99
Blood loss [ml]	5	156	140	17.41 (−23.18, 58.00)	0.4	7.96	4	50	0.09
Operation time [min]	5	156	140	7.24 (−4.04, 18.52)	0.21	12.17	4	67	0.02

Abbreviations: df, degree of freedom; WMD/OR, weighted MD/OR. *P<0.05

**Figure 13:**
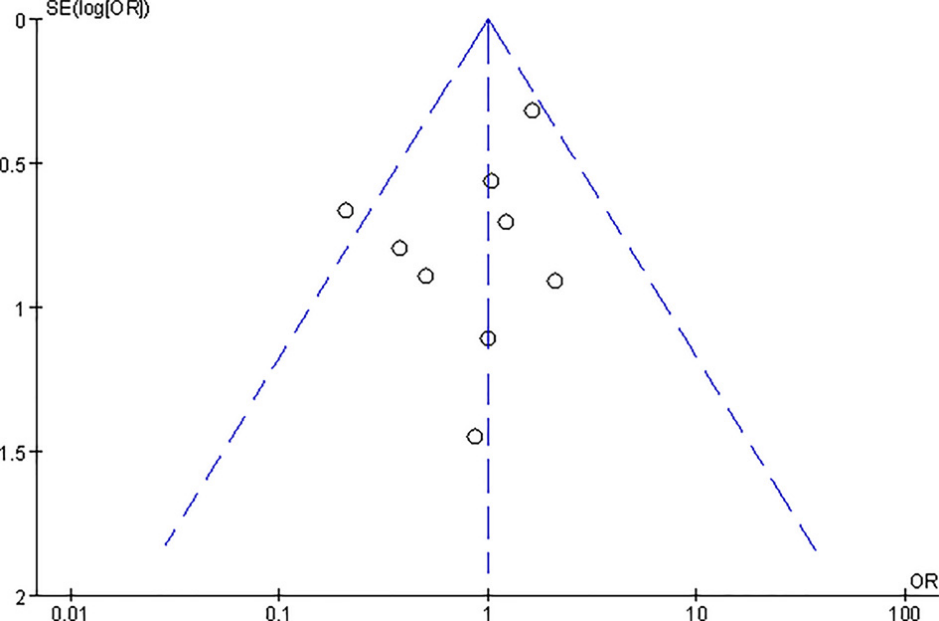
Funnel plot which was illustrated by non-union rate shows the publication bias

Publication bias was evaluated by the funnel plot (Figure 13), which described the complication rates between two groups. A total of nine studies almost lie inside the 95% CI and the distribution was symmetrical, which indicated no obvious publication in this meta-analysis.

## Discussion

Reduction in surgical management of patients with lumbar spondylolisthesis remains controversial. Several trials studied the correlation of clinical outcomes and reduction during surgical procedure [18]. However, few studies with sufficient sample size evaluated it. Therefore, this study assessed clinical and radiological results by meta-analysis.

In this article, the quality of eight retrospective articles were evaluated by modified NOS, and other two RCTs were defined as high-quality studies directly. The modified NOS was different from original one in comparability and outcomes. It is possible that the preoperatively basic characteristics, including clinical and radiological parameters, of patients affected the outcomes. Therefore, the comparability 3 stars, which contained Age, VAS score, ODI score, JOA score, Radiological parameters, and Meyerding grade was scored before surgery. The outcome and exposure were scored two points here. Considering that the outcomes not only included short-term items, such as blood loss, hospital stay, operative time, et al. but also contained much more long-term indexes, the length of follow-up was not important for our current study. So the assessment criteria that if it was enough follow-up for outcomes to occur in outcome and exposure was eliminated in our study.

Primary outcomes showed that the union rate was significantly higher in reduction group comparing to fusion *in situ* group, which was consistent with the previous studies [18]. These results probably suggested that reductive vertebrae could provide more solid reconstruction and contact area, leading to a better fusion. However, other clinical criteria, including VAS score, ODI score, JOA score, and satisfaction, had no statistical differences. These findings were consistent with the widely spread opinion that neurological decompression and vertebrae fusion are the main aims of surgery [4]. Surgical technique with or without reduction all achieved these goals. Considering that the pooled data in the present study were mostly from the reports describing patients for short-term follow-up, different clinical outcomes might be obtained due to the different union rate between the reduction groups and the *in situ* groups after long-term follow-up.

Previous study indicated that more patients suffered from neurological symptoms in reduction group, which was also considered as an important disadvantage during reductive procedure [4]. Nevertheless, in our analysis, the occurrence rate of complication was similar in two groups. This might be because the spinal canal and nerve roots far distally and laterally were decompressed before slip reduction, permitting complete visualization of the roots at all times [4]. In addition, there was no statistical differences between these two groups in blood loss and operative time. This might be due to the development of modern surgical instrumentation and techniques.

Our study demonstrated that slippage was significant improved in reduction group, while lumbar lordosis and lumbosacral angle were similar in both groups no matter before or after surgery. These results provided less meaningful evidence to sagittal plane for stable balance. Some significant spinopelvic parameters, such as pelvic incidence (PI), sacral slope (SS), pelvic tilt (PT), etc, could not be synthesized for not enough data in these articles.

The treatments for different slippage grades were different, especially moderate and severe ones. We divided spondylolisthesis into moderate (Meyerding grades I, II) and severe slippage (Meyerding grades III, IV) to do the subgroup analysis. All these results were similar to original outcomes. From these subgroup analysis, we concluded that the treatment selection was similar in slight or high-grade spondylolisthesis.

There was nearly no heterogeneity changes in subgroup analysis comparing with original outcomes. This indicated that the Meyerding grade of spondylolisthesis may not responsible for the heterogeneity. However, considering that the heterogeneity of criteria we brought into subgroup analysis were generally low, more articles were needed to compare outcomes between two surgical methods in different grade spondylolisthesis.

The most common reasons that caused spondylolisthesis were spondylolysis and degeneration. The treatments for these two types of spondylolisthesis might be different. In order to explore the treatment differences, isthmic and degenerative spondylolisthesis were analyzed separately. Due to the quantitative limitation of articles which described degenerative spondylolisthesis, only articles which investigated isthmic spondylolisthesis were extracted. The outcomes were no difference for the type of spondylolisthesis possibly. Nevertheless, more trails of degenerative spondylolisthesis should be further studied.

The sensitivity analysis was performed in seven high-quality articles (two RCT and five high-quality retrospective studies) in order to improve the credibility of our study. All these articles matched the basic characteristics of patients in two treatment groups before surgery. The results were almost consistent with original ones. Meanwhile, heterogeneity was generally lower in each item. Low-quality articles may contribute to the occurrence of heterogeneity.

Certainly, there were several limitations in our study. First, although the quality of seven articles in our meta-analysis were high, only two RCTs were brought into our analysis. Second, the heterogeneity was a little high in some indexes. Despite the heterogeneity was not significant for dichotomous outcomes, it was significant for most of the continuous variables, including studies, researched in different countries, different operative methods, different slippage degree, and measurement of outcomes. These differences might contribute to the significant heterogeneity. Pooling of data using the random-effects model might reduce the effect of heterogeneity but could not abolish it.

In conclusion, both reduction and fusion *in situ* for lumbar spondylolisthesis were related with good clinical results. Reduction leaded to higher rate of fusion, better radiographic slippage, and shorter hospital stay. After sufficient decompression, reduction did not incur additional risk of neurologic impairment compared with fusion *in situ*.

## Abbreviations

MIS-TLIF: minimally invasive transforaminal lumbar interbody fusion
NOS: Newcastle–Ottawa Scale
OR: odds ratio
RCT: randomized controlled trial
TLIF: transforaminal lumbar interbody fusion
95% CI: 95% confidence interval
